# The Power of Assemblies at Interfaces: Nanosensor Platforms Based on Synthetic Receptor Membranes

**DOI:** 10.3390/s20082228

**Published:** 2020-04-15

**Authors:** Tsukuru Minamiki, Yuki Ichikawa, Ryoji Kurita

**Affiliations:** 1 Biomedical Research Institute, National Institute of Advanced Industrial Science and Technology (AIST), 1-1-1 Higashi, Tsukuba, Ibaraki 305-8566, Japan; cityyukirin142563@gmail.com; 2 DAILAB, DBT-AIST International Center for Translational and Environmental Research (DAICENTER), National Institute of Advanced Industrial Science and Technology (AIST), Central 5-41, 1-1-1 Higashi, Tsukuba, Ibaraki 305-8565, Japan; 3 Faculty of Pure and Applied Sciences, University of Tsukuba, 1-1-1 Tennodai, Tsukuba, Ibaraki 305-8573, Japan

**Keywords:** molecular recognition, chemical sensors, molecular assemblies, multivalent interactions, Langmuir–Blodgett films, self-assembled monolayers, artificial receptors, surface plasmon resonances, field-effect transistors, surface analyses

## Abstract

Synthetic sensing materials (artificial receptors) are some of the most attractive components of chemical/biosensors because of their long-term stability and low cost of production. However, the strategy for the practical design of these materials toward specific molecular recognition in water is not established yet. For the construction of artificial material-based chemical/biosensors, the bottom-up assembly of these materials is one of the effective methods. This is because the driving forces of molecular recognition on the receptors could be enhanced by the integration of such kinds of materials at the ‘interfaces’, such as the boundary portion between the liquid and solid phases. Additionally, the molecular assembly of such self-assembled monolayers (SAMs) can easily be installed in transducer devices. Thus, we believe that nanosensor platforms that consist of synthetic receptor membranes on the transducer surfaces can be applied to powerful tools for high-throughput analyses of the required targets. In this review, we briefly summarize a comprehensive overview that includes the preparation techniques for molecular assemblies, the characterization methods of the interfaces, and a few examples of receptor assembly-based chemical/biosensing platforms on each transduction mechanism.

## 1. Introduction

Molecular recognition systems in organisms contribute to the tuning of biological functions [1]. For instance, enzymes which have substrate specificity can catalyze highly efficient and selective biochemical reactions under ambient temperature and pressure conditions. Similarly, transfer of genetic information from parents to offspring takes place through the formation of self-organized double-helix structures called deoxyribonucleic acids (DNAs) [2]. Although most of the chemical driving forces at the center of these biological functions are weak noncovalent interactions (e.g., hydrogen bonding, electrostatic interactions, etc.), the assemblies of various functional molecules in a biological system can accomplish the construction of a specific “field” toward selective molecular recognition. Hence, if we can imitate such systems, molecular-assembly approaches can be helpful in developing high-performance artificial sensor systems without delicate design and complicated processes of functional materials [3].

**Figure 1:**
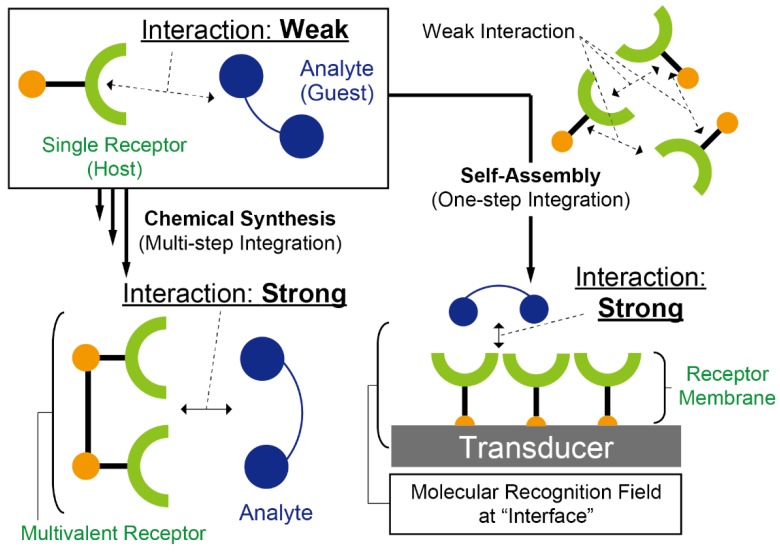
Schematic concept of molecular assembly-based artificial receptor systems for sensing applications. Although the wiring of receptor molecules is an effective strategy to improve molecular interactions between the host receptors and guest analytes, the complicated multi-step process is required for the preparation of the multivalent receptor by using chemical synthesis processes. In contrast, the one-step preparation of the multivalent receptors at the transducer interface can be easily achieved by molecular self-assembly. Hence, the precise design of sensing materials and the expression of their functionality can be effectively achieved by imitating the molecular recognition systems in nature.

Synthetic receptors (i.e., artificial molecular recognition materials) are some of the most suitable platform materials for the development of chemical/biosensors owing to their chemical/physical stability, low cost of production, and their fine-tuning ability to select the required targets [4]. However, the preparation of artificial receptor-based sensors for practical applications is still in its initial stages, since the analyte specificity of most of the artificial materials is generally lower than that of biomaterials (i.e., antibodies and enzymes) [5]. To improve the binding affinity between the artificial receptors and the analytes, complicated synthesis procedures are required. Hence, a simpler way to improve the sensing ability of the artificial receptors should be considered to bring out the attractive features of these materials. To facilitate this, bottom-up integration of the receptors as molecular assemblies is considered as one of the most useful approaches for the construction of selective sensing fields for analytes. Moreover, the sensing features in the artificial receptor-based sensors could be finely tuned by using top-down technologies (e.g., molecular imprinting techniques [6], etc.) because the molecular recognition ability of the receptor assemblies depends on their nanostructures [7]. As aforementioned, the molecular assembly enhances the functions of a single kind of molecule (Figure 1) [8]. In addition, the driving forces in molecular recognition (i.e., noncovalent interactions) can be amplified at the interfaces such as the boundary portion between the liquid and solid phases [9,10]. Thus, we believe that the installation of artificial receptors at the surface of the sensing portion in selective transducers is an effective approach to prepare artificial receptor-based sensor systems.

In this review, we briefly summarize a research overview including the construction methods of molecular assemblies, the characterization techniques for the interfaces, and the rational design and demonstrative examples of “nanosensors” (i.e., chemical/biosensing platforms based on synthetic receptor assemblies) utilizing each transduction mechanism.

## 2. Installation of Molecular Assemblies at the Interfaces

This section introduces representative methods for the installation of molecular assemblies at the interfaces. In general, the sensing ability of single-molecule receptors in a bulk aqueous phase is lower than that of naturally-derived molecular recognition materials because the molecular interactions between the receptors and the analytes are strongly affected by their hydrations. This means that any extractions of analytes from the target medium in the presence of various interferents by using organic solvents are generally required for the achievement of selective detection of analytes in artificial receptor-based sensors in practical applications. In contrast, the interface microenvironments enable the enhancement of the molecular recognition ability of synthetic receptors because the hydration effect can be reduced at the interfaces. This is possible because the interfacial dielectric constant is much lower than in bulk water [11]. Furthermore, the orderly arrangement of receptor molecules at the interfaces causes multivalent interactions between the receptors and the analytes (Figure 1) [12]. Hence, the receptor assemblies formed at the interfaces could accomplish the challenging topic of increasing the sensing ability of single-molecule receptors. In this regard, the techniques proposed in this section for the construction of molecular assemblies have popularly been employed for the development of chemical/biosensors, based on synthetic receptors.

### 2.1. Functionalization of Interfaces by Using Langmuir–Blodgett Films

**Figure 2:**
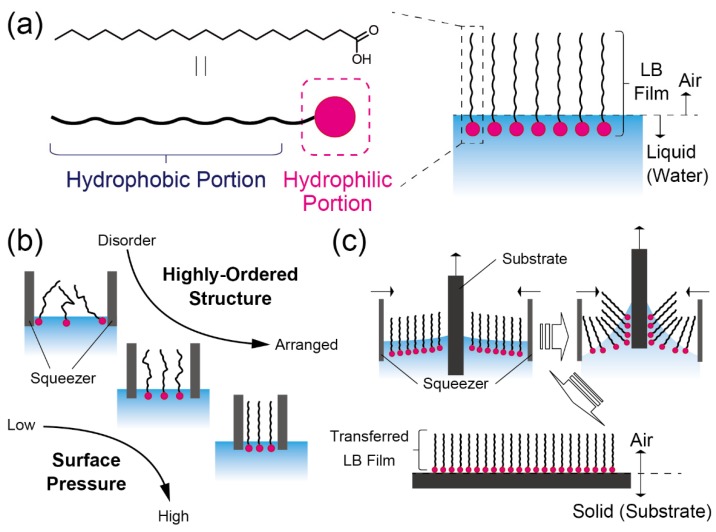
(**a**) Schematic illustration of a component material for the preparation of an LB film. A chemical structure indicates the typical molecule of the component (stearic acid). (**b**) The different phases of the LB films at each surface pressure. (**c**) The transfer procedure of the LB film from water to the substrate surface.

Langmuir–Blodgett (LB) films have been investigated earlier for both fundamental material properties and practical applications in nanotechnology [13]. In this approach, mono- or few-molecule layers can be assembled by the layer-by-layer process [14]. The design of materials and the assembly processes of the LB films are inspired by the supramolecular structures of lipid bilayer membranes in organisms. The component materials of LB films consist of intramolecular-connected segments, which are both hydrophobic and hydrophilic units (Figure 2a). Such amphiphilic organic compounds are introduced and assembled as molecular membranes at the interfaces. When these amphiphilic compounds are dissolved in water, the molecules congregate on the liquid surface (at the water–air interface) or the liquid–liquid interface (at the boundary portion between the polar and nonpolar solvents). Thereby the molecules are assembled like a thin film at the interfaces. During this process, the hydrophobic region of the molecules is exposed to the air phase, and the hydrophilic region is oriented towards the water phase. Thereafter, the highly-packed molecular film can be prepared by applying a constant surface pressure at the water–air interface using squeezers (Figure 2b). Thus, the molecular density of the components in the LB film can be systematically controlled by changing the applied surface pressure. To trap the analytes contained in the water at the water–air interface, receptor moieties should be conjugated to the hydrophilic region in the component molecule of the LB monolayer [15,16,17].

To obtain the functionalized substrate, the LB film should be separated onto a solid surface from the liquid phase. Initially, the substrate is immersed perpendicularly into the water containing the LB molecules. Then, the LB film is successfully transferred onto the solid surface from the water by raising the substrate from the solution carefully (Figure 2c). The transferred LB film is noncovalently adsorbed onto the substrate, which means that the film has not anchored onto the solid surface. This enables the highly-ordered structures in the LB film to be reorganized and rearranged by applying physical or chemical stimulation. This renders the functionality of the LB film to be dynamically modulated [18]. However, this mechanical flexibility prevents the usage of the LB film for practical applications because it is difficult to assure reproducibility and the stability of these films.

### 2.2. Decoration of Solid Surfaces with Self-Assembled Monolayers

**Figure 3:**
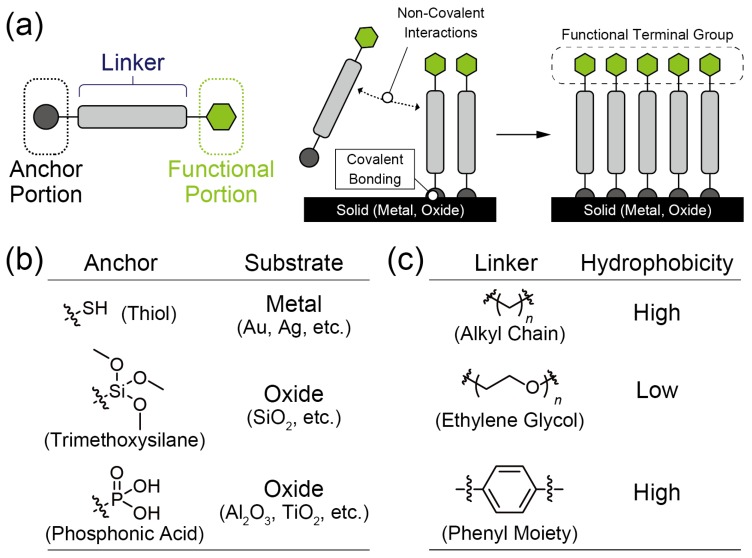
(**a**) Schematic representation of a SAM formation. (**b**) Example groups of the anchor portion in SAM components for the modification on various surfaces. (**c**) Typical structures of the linker portion in the SAM with the different hydrophobicity.

Spontaneously formed molecular monolayers at solid surfaces are called self-assembled monolayers (SAMs) [19]. In general, SAM molecules are composed of three parts: (1) a head group for anchoring SAM molecules onto the substrate, (2) a linker portion, and (3) a terminal group as the functional portion (Figure 3a). Initially, the SAM molecule adsorbs onto the solid substrate (e.g., metal, metal oxide, etc.) through covalent bonding. For example, chemisorption between gold (Au) surfaces and thiol compounds has been well studied in the SAM research because the thiol materials can strongly react with the Au substrate (Figure 3b) [20]. While almost-SAMs have been reported for the decoration on metal/oxide surfaces, the surface treatment of polymer substrates has also been investigated [21]. This suggests that the SAM can functionalized more universal sensing platforms such as polystyrene-based microtiter plates. After the adsorption of these molecules onto the solid surface, the attached molecules can naturally organize into a well-ordered and packed film structure. This is attributed to the molecular interactions among the linker portion in the SAM compound, such as the hydrophobic interaction and π–π stacking (Figure 3c). Consequently, the functional portion of the SAM molecule assembles on the substrate surface methodically. Hence, the SAM formation can easily enable the substrate to be used for the desired functions. Although this formation can be achieved in either the vapor or the liquid phase, a slight difference arises in the ordered structures in the molecular layer due to the varying conditions that exist during the SAM formation [20]. Hence, we need to pay attention to the conditions (e.g., concentration, temperature, processing time, etc.) for preparing reproducible surfaces. Interestingly, it is possible to pattern the SAM films by using general microfabrication techniques [22]. Thus, the desired functions can selectively be implemented at the arbitrary regions on the devices. Since the functionalization of the solid surface by the SAMs is easier and more reproducible than the method of transferring LB films (vide supra), SAM techniques have been widely utilized for various applications including the development of chemical/biosensors [23].

## 3. Characterization Methods for Molecular Assemblies at the Interfaces

To confirm the installation of molecular assemblies for each sensor, characterizations of the material-functionalized interfaces at the sensing portion should be performed. Since there are many evaluation criteria of the molecular assemblies at the interface (e.g., hydrophobicity, molecular density, composition elements, etc.), various analyses for the assemblies from diversified standpoints are required. In this section, standard techniques for the characterization of synthetic receptor membranes in the sensor platforms are described.

### 3.1. Contact Angle Goniometry

**Figure 4:**
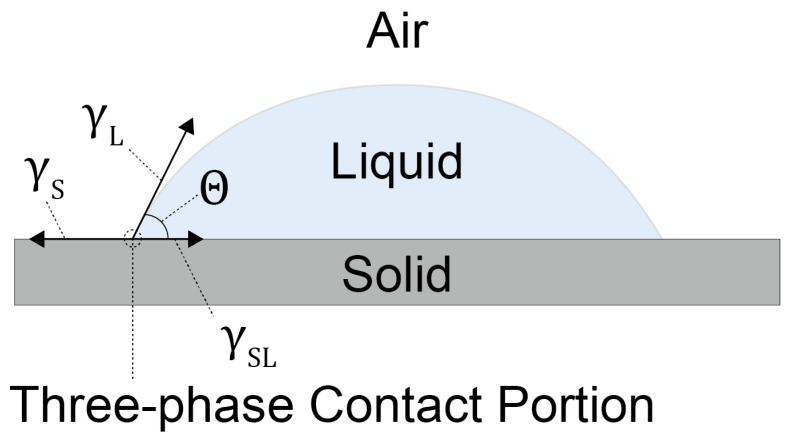
A schematic diagram of a dropped liquid on a solid surface. The arrows at the three-phase contact portion indicate each interfacial tension.

Contact angle goniometry (CAG) analysis is the most utilized method to confirm the formation of molecular assemblies at the interfaces because it is easy to use and is rapid [24]. Contact angles of the liquid droplet on the molecular membrane are reflected by the surface energy of the substrate, which means that the CAG technique can evaluate the hydrophobicity and hydrophilicity of the surface of the molecular assemblies. In general, the wetting properties on the surface of the substrates are investigated by using a water droplet. When the water droplet contacts the surface, a three-phase contact line (the wetting line) is shaped at the edge of the droplet (Figure 4). Based on this contact line, the interfacial tensions can be estimated by using the following Equation (1) (Young’s equation) [24].

(1) $$\gamma_{L}\cdot \cos\theta =\gamma_{S}-\gamma_{\mathrm{SL}}$$

Here, *γ*_L_, *γ*_S_, and *γ*_SL_ are the interfacial tensions at the liquid surface, the solid surface, and the liquid–solid interface, respectively. *Θ* is the contact angle, which consists of the three-phase contact line (Figure 4). From this equation, we can find the contact angle, which directly reflects the hydrophobicity of the solid surface [25]. To enhance the intermolecular forces between the component molecules in the molecular assembly, hydrophobic moieties are generally incorporated into the components [26]. In contrast, the contact angle is strongly affected by the hydrophobicity and hydrophilicity of the terminal groups in the assembly. Considering these aspects, we note that the CAG method can only be applied for the qualitative investigation of the assembly, as the contact angle indicates the macroscopic information at the solid surface [27].

### 3.2. Elemental Analyses of Molecular Assemblies

**Figure 5:**
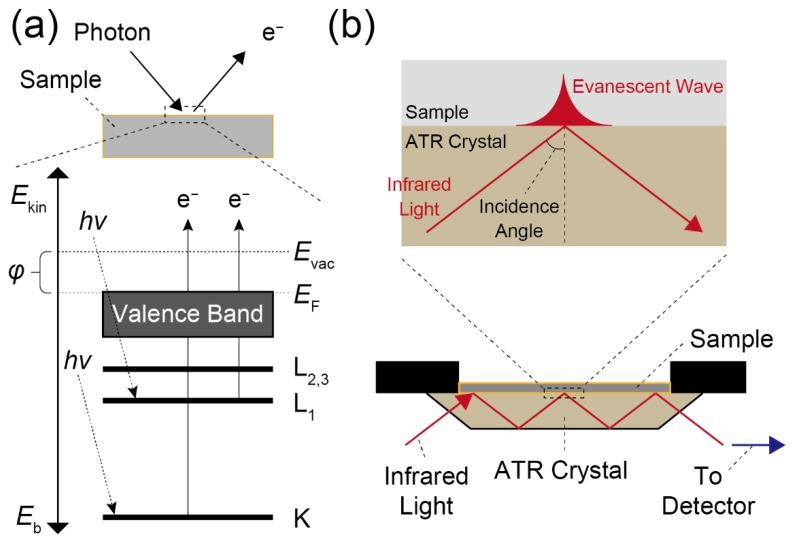
(**a**) Schematic energy diagram in the XPS measurement. *φ* indicates the work function of the sample. (**b**) Illustration of a multiple-reflection type ATR system.

To determine the elemental composition of the molecular assembly formed at the interfaces, the molecular information can be chemically analyzed using X-ray photoelectron spectroscopy (XPS) or electron spectroscopy for chemical analysis (ESCA) [24]. When the substrate surface is exposed to monochromatic photons at high energy (>1 keV), the photons excite the atoms of the installed molecules. Therefore, the kinetic energy of the emitted photoelectrons can be determined by these measurements. In the XPS measurement, the relationship between the photon and the kinetic energy can be provided by the following Equation (2) [24]:

(2) $$E_{b}=E_{\mathrm{photon}}-\left\{E_{\mathrm{kin}}+\left(E_{\mathrm{vac}}-E_{F}\right)\right\}$$

where *E*_b_, *E*_photon_, *E*_kin_, *E*_vac_, and *E*_F_ are the binding energy of the electron, the energy of the X-ray photons (=*hν*), the kinetic energy of the electron as measured values, the vacuum energy, and the Fermi energy, respectively (Figure 5a). Herein, the kinetic energy depends on the binding energy of the electron in the target molecule, which means that the results obtained from XPS or ESCA show the chemical bonding states of the molecule. Hence, XPS and ESCA are the standard techniques used in surface analysis. For instance, XPS can not only characterize the elemental composition of SAMs, but also the highly-ordered structures in the SAMs which can be assumed from the XPS spectra [28,29]. 

Although these analyses have high sensitivity and resolution, samples that can be evaluated by XPS and ESCA are limited to the solid substrate because these analyses should be performed under vacuum. In this regard, it is also possible to characterize the elemental components in the molecular assembly using Fourier transform infrared spectroscopy (FT-IR) [30]. The elemental analysis of the molecular assemblies installed at various interfaces, including the LB film on the liquid, can be achieved by FT-IR, as the measurements can be performed under atmospheric conditions. When the infrared light of the wavelengths between approximately 780 nm and 50 µm (12,800–200 cm^−1^) irradiates the molecules, the incident light is absorbed at a specific wavenumber due to the vibration and/or stretching of the target molecules in accordance with the chemical bonding state in the compounds. Thus, the FT-IR measurement can evaluate the chemical information of the molecules as with the abovementioned elemental analyses. The attenuated total reflection (ATR) method is one of the popular methods to perform the interfacial analysis based on FT-IR. In the ATR method, the chemical information at the interface can be easily obtained by measuring the total reflection beam from the sample (Figure 5b). While the sensitivity and resolution of the FT-IR technique are relatively lower than those of the photoemission-based spectroscopies (i.e., XPS and ESCA), the FT-IR measurement has been widely employed for elemental characterization of the molecular assemblies formed at the interfaces, owing to its simplicity and versatility [30].

### 3.3. Direct Observation of the Assemblies Installed at the Interfaces

**Figure 6:**
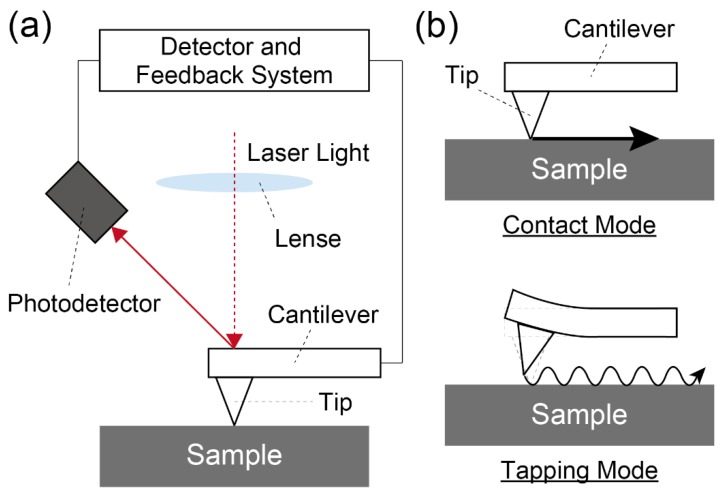
(**a**) Schematic representation of an AFM measurement setup. (**b**) Example illustrations of each measurement mode of the AFM.

Since the function of molecular assemblies follows their macro and microscopic structures [31], direct observation for the interfaces is crucial to gain a deeper understanding of the installed assemblies. In this regard, an atomic force microscope (AFM) and scanning tunneling microscope (STM) are commonly utilized to analyze the stereoscopic structures on the interfaces [24]. While the basic principle of these types of microscopes is very similar, the AFM is more widely utilized for the direct observation of the interfaces because the measurable samples on the STM are much less than those on the AFM measurement. In these methods, a tiny tip attached to a micro-cantilever scans the sample surface as tracing the surface of the steric structure. The edge of the tip softly touches on the object surface, and then the sample stage is moved by a slightly movable piezoelectric scanner. After that, the displacement magnitude of the tip, followed with the stereoscopic structure on the surface is detected by reflected light from the upper side of the cantilever. In this way, a topographic image of the sample surface is acquired by the AFM measurement (Figure 6a). Although the elevation of the pushing depth of the tip onto the sample surface can improve the resolution of the object image, the direct approach of the tip to soft or deformable samples (e.g., LB films) disfigures the highly-ordered structures in such surfaces. To avoid the deformation of the microstructure at the object surface, the tapping mode is utilized in the AFM measurement for fragile samples (Figure 6b). In the tapping mode, a vibrated cantilever approaches the sample surface. When the tip contacts the object, the amplitude of the vibrated cantilever decreases. Thus, the acquirement of the topographic image can be achieved by mapping the obtained reduction of the amplitude. The AFM measurement exhibits excellent resolution with the range of the molecular level; therefore, the AFM method is one of the useful techniques for the direct observation of the three-dimensional structures on the molecular assemblies.

### 3.4. Determination of Electrical Potential at the Interfaces

Molecular assemblies formed in an orderly line at the interfaces strongly influence interfacial electrical potentials (e.g., the work function of metals modified with SAMs). This is attributed to the dipole moment of the arranged molecules in the assemblies [32]. Therefore, electrical properties of the installed molecular assemblies at the interfaces can be discussed by determination techniques for the interfacial potentials. From this point, UV photoemission spectroscopy (UPS) is one of the effective techniques for the characterization of the molecular assemblies [33]. The UPS measurement is performed pursuant to the outer photoelectron effect [24]. In this method, atoms and molecules at the surface are ionized by using photons with energies between approximately 10 and 100 eV. The threshold energy for the emission of electrons from the surface is equivalent to the ionized potential of target materials. Hence, the electrical properties of the installed assemblies can be characterized by measuring the surface before and after the installation of the assemblies. While the UPS shows the high sensitivity for the determination of interfacial potentials, the measurable sample is restricted to the solid surface, since the UPS should be carried out in vacuum conditions.

In contrast, measurement samples on photoemission yield spectroscopy (PYS) are not confined in solid materials because PYS with an open counter can perform under atmospheric conditions [34]. In fact, Minamiki et al. demonstrated the in situ evaluation for the electrical potential of the synthetic receptor membrane upon bonding with the target protein by using PYS [35]. To analyze the more topical information of the assemblies, the kelvin force microscope (KFM) is also used for the measurement of electrical potential at the sample surface [36]. Because the basic principle of the KFM measurement is similar to the AFM method, the direct observation of potential mapping at the interfaces can be obtained by using this technique, owing to its excellent resolution. Notably, the obtained electrical potential of the assemblies from these measurement techniques is very crucial to achieve the effective material design for electrochemical sensors (vide infra).

### 3.5. Characterization of Decorated Interfaces by Other Methods

More individual techniques have been applied for the characterization of substrate surfaces. A fluorescent labeling technique is one of the simple tools to observe mesoscopic structures on the substrate surfaces [37]. The interfacial capacitance of the SAMs is determined by using electrochemical impedance spectroscopy (EIS), which is one of the electrochemical measurement methods [38]. Furthermore, spectroscopic ellipsometry can directly measure the thickness of ultra-thin films, such as molecular monolayers [39]. Even apart from the presented techniques in this review, molecular assembly-decorated interfaces can be characterized by using various analysis techniques [24].

## 4. Synthetic Receptor Membrane-Based Sensing Platforms for Chemical Bio-Analyses

To develop chemical/biosensing platforms based on synthetic receptor membranes for practical applications such as environmental assessment and diagnoses, the combination of receptor assemblies with platforms is required to retrieve the molecular recognition at the interfaces. Herein, we summarized various types of chemical/biosensors that consist of artificial receptor membranes suitable for each transduction mechanism (Table 1). The sensing performances (e.g., sensitivity, quantitativity, selectivity, etc.,) not only depend on the molecular recognition ability of artificial receptors but also the utilized transduction mechanism, especially when the detectable size of analytes on each transducer is different. In this regard, we summarize the “typical” properties of the presented transduction mechanisms in this review (Table 2). Notably, the mentioned properties in Table 2 are often varied, even if we used the same transduction mechanism. More importantly, the sensing performances can be tuned by altering the applied artificial receptors. The concrete examples of each combination of artificial receptors and transduction mechanisms are summarized in this chapter.

### 4.1. Colorimetry or Fluorometry-Based Sensors

To perform chemical sensing based on synthetic receptor membranes, colorimetric or fluorometric approaches have been widely studied, owing to their simplicity and rapid detection quality [56]. To achieve “visualization” of the target information, the conjugated moiety (i.e., dyes and/or fluorophores) should be attached to the sensing membranes.

**Figure 7:**
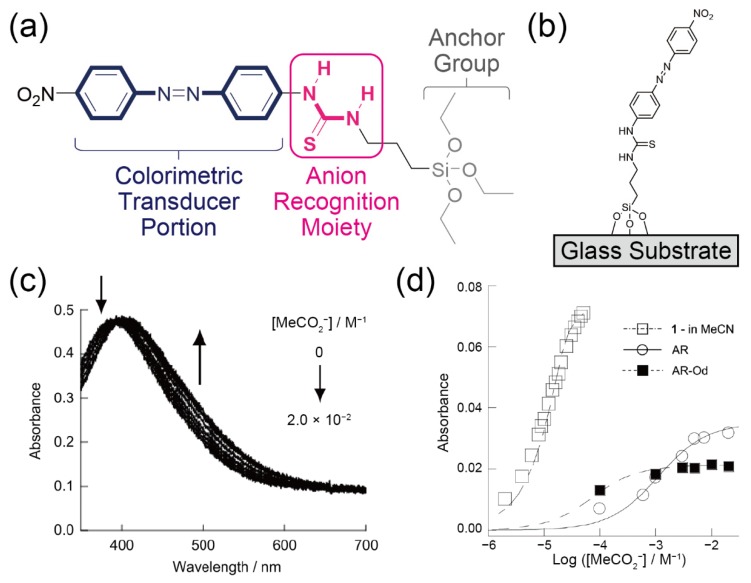
(**a**) Chemical structure of a *N*-(4-(4-nitrophenylazo)phenyl)-*N*′-propyl (NPPP) derivative for the SAM-based colorimetric sensor. (**b**) Surface structure of the NPPP-SAM. (**c**) Changes in the absorption spectra of the SAM-modified glass upon the addition of the acetate anion in acetonitrile (MeCN). (**d**) Changes in the absorbance of each sensor for various levels of acetate in MeCN. The indicated sensors were the NPPP molecule in the MeCN solution (=**1**-in MeCN), the NPPP-based anion receptor film on the glass (=AR), and the NPPP-SAM formed with an octadecyl monolayer (=AR-Od). Reproduced and adapted with permission from reference. [40]. Copyright 2013 The Royal Society of Chemistry.

For instance, the colorimetric detection of anions (CH_3_CO_2_^−^, H_2_PO_4_^−^, Cl^−^) based on a thiourea monolayer was reported [40]. To realize the optical detection of analytes, *N*-(4-(4-nitrophenylazo)phenyl)-*N*′-propyl moiety (NPPP) was utilized as the colorimetric transducer for the molecular assembly (SAM)-based sensor (Figure 7a). To form the SAM onto the glass substrate, tri(ethoxysilyl)-terminated NPPP-thiourea was initially synthesized. Then, the glass surface was treated with the prepared compound (Figure 7b). To evaluate the sensing ability of the synthesized receptor, the anion recognition behavior of the non-immobilized receptor was examined in an organic solvent (acetonitrile) by UV–Vis spectroscopy. The resulting color changes were observed upon the addition of anions into the receptor-dissolved solution. This indicates that the complexation of the thiourea portion of the receptor molecule and the target anions induces the electron transfer phenomenon from the chromophore (NPPP) [57]. This mechanism of color change of the NPPP-thiourea was also supported by a study on nuclear magnetic resonance (NMR). Thereafter, the titration experiment of anions on the SAM-coated glass was carried out. While the absorption spectra of the glass changed in the range of mM order with increasing MeCO_2_^−^ concentration, the spectra of acetonitrile containing the same analyte and the non-immobilized receptor were shifted in the range of µM order (Figure 7c). This reduction of the sensing signal might be derived from the higher-ordered structure of the SAM. When the molecular film was constructed only using the silane-terminated NPPP-thiourea, there is not enough space for anion sensing at the thiourea portion. In fact, the sensitivity of the colorimetric sensor was slightly improved by applying the SAM of the NPPP-thiourea mixed with an alkyl-silane agent (Figure 7d), supporting the fact that the changes in the nanostructures (i.e., the intermolecular distance of the anion recognition moiety) of the receptor assembly might affect the sensing ability of these kinds of sensors.

**Figure 8:**
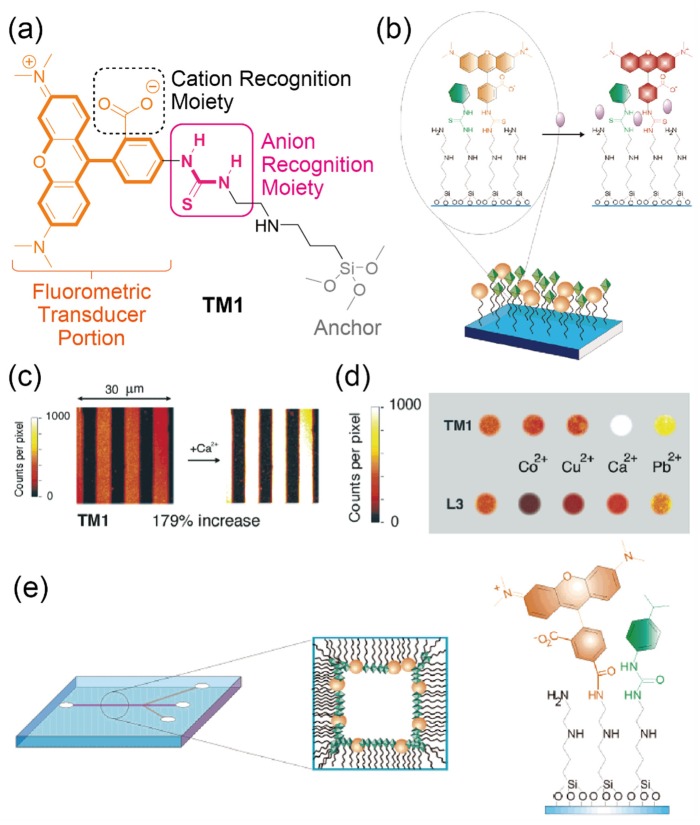
(**a**) Chemical structure of the TAMRA-attached SAM (**TM1**) for fluorometric sensors. (**b**) Schematic illustration of the fluorescent SAM. (**c**) Confocal fluorescence microscopic images of the patterned SAM before and after dipping into the Ca^2+^ solution. (**d**) Fluorescence images of a microarray based on the patterned SAMs. (**e**) Representation of the SAM-modified microfluidic channel. Reproduced and adapted with permission from reference [41]. Copyright 2004 American Chemical Society.

Although it is possible to achieve simple discrimination of the analytes using colorimetric sensors, fluorometric approaches can improve the sensitivity and the quantitativity of molecular assembly-based optical sensors. Crego-Calama et al. demonstrated the fluorometric detection of analytes by using thiourea and carboxy-functionalized SAMs [41]. Notably, the receptor design for anions is almost the same as that of the abovementioned colorimetric-type sensor. 5-Carboxytetramethylrhodamine (TAMRA, **TM1**), tetramethylrhodamine (TRITC), or lissamine (**L3**) fluorophores were employed to achieve fluorometric detection of analytes on the glass substrate (Figure 8a,b). These fluorophores and different binding moieties for analytes were immobilized to an amine-terminated SAM (*N*-[3-(trimethoxysilyl)propyl]ethylenediamine; TPEDA) on the substrate. The fluorescence intensity in the prepared SAMs immobilized with various molecular components increased/decreased upon the addition of each analyte. The versatile fluorescence-quenching phenomenon seen could not only be derived from the intermolecular bonding between the individual fluorophores and analytes but might also be affected by changing the highly-ordered structures of the SAMs induced by the analyte recognition at the sensing portions. The analyte selectivity of the SAMs reflected the molecular recognition ability of each sensing moiety. Importantly, the sensitivity in the SAM-based fluorometric sensors was much higher than that of in the colorimetric sensors (Table 1). To achieve high-resolution microsensors based on the fluorometric SAMs, the micropatterning of the SAMs was also demonstrated by using microcontact printing (µCP) (Figure 8c,d). The results obtained from the micropatterned sensors were similar to the large-sized substrate functionalized with the same SAMs, indicating that the micropattern techniques for the SAMs can contribute to the fabrication of microsensor array platforms for high-throughput chemical analyses. Furthermore, the incorporation of the SAMs into a microfluidic device was successfully performed in this study (Figure 8e). The fabricated microdevice modified with the fluorometric SAMs responds to the continuous changes in the analyte concentrations. This suggests that the prepared device could be applied for real-time monitoring of the analyte levels. In addition, the microspaces can dramatically amplify the efficiency of chemical reactions [58]. Hence, improved sensitivity of assembly-based sensors might be achieved by using microfluidic devices.

### 4.2. Transduction of Sensing Information Based on “Invisible” Changes in Optical Signals: Toward the Development of High-Performance Sensors

As described above, the fluorometric transduction of chemical information of the captured analytes on the molecular assemblies is a simple way to construct chemical sensors. However, the sensitivity of the introduced sensors is generally lower than that of conventional fluorometric probes. In this regard, “invisible” changes in optical signals have been applied for the improvement of the sensitivity in the molecular assembly-functionalized platforms.

**Figure 9:**
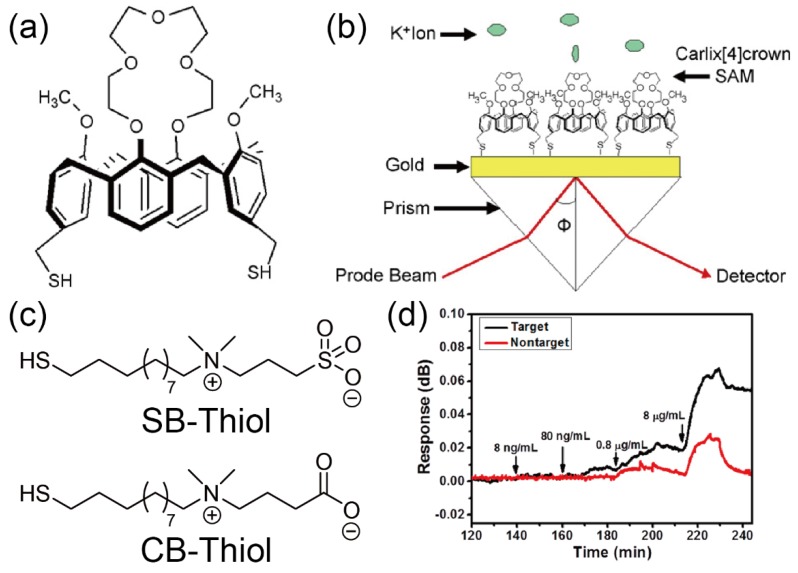
(**a**) Chemical structure of the calix[4]crown derivative for the preparation of SPR sensors. (**b**) Schematic illustration of a calix[4]crown SAM-modified SPR sensor for the detection of K^+^. Reproduced and adapted with permission from reference [42]. Copyright 2008 Elsevier B. V. (**c**) Molecular structures of the zwitterionic compounds for the construction of the SAM-based protein sensor. (**d**) Changes in the SPR responses of target (rabbit polyclonal IgG) and nontarget (chicken polyclonal IgG) onto the mixed SAM by flowing secondary antibodies (goat anti-rabbit IgG) at various concentrations. Reproduced and adapted with permission from reference [43]. Copyright 2019 American Chemical Society.

Surface plasmon resonance (SPR) methods are popular approaches for the enhancement of optical signals from synthetic receptor membranes installed at the interfaces [59]. SPR is the resonant oscillation phenomenon of electrons at the interface between the metal substrates and the analyte medium (liquid or air) by incident light. When the target molecules are captured on the metal substrate, the SPR signal is drastically changed, which depends on the permittivity of the captured molecules. This means that the SPR devices can sensitively reflect the molecular recognition behavior of the sensing material-activated metal surfaces. Therefore, the SPR methods have widely been applied for the construction of bio/chemical sensors. For example, Koh et al. successfully demonstrated the sensitive detection of potassium ion (K^+^) by using the SPR sensor functionalized with a calix[4]crown SAM, which has the specific sensing ability for K^+^ (Figure 9a,b) [42]. In general, although SPR sensors can sensitively respond to the addition of macromolecular targets (e.g., proteins), it is difficult to detect small molecules [59]. However, the SAM-modified SPR device showed high selectivity and sensitivity for the small-sized target (K^+^) (detection limit: 1 pM), suggesting that the combination of the SPR sensors and the synthetic receptor membranes can synergistically boost the sensing ability of each other. The protein detection studies based on the SAM-modified SPR sensor were also carried out by Huang et al. [43]. In these studies, mixed zwitterionic SAMs (i.e., anionic carboxybetaine (CB) and cationic sulfobetaine (SB)) were utilized as the protein recognition membranes. The control of the electrostatic interactions between the SPR surfaces and the target proteins (IgG) was systematically achieved by modulating the pH-driven intermolecular complexation which consisted of the charged terminal moieties in the SAM (Figure 9 c,d). Furthermore, the SPR devices are some of the suitable platforms for the development of portable chemical/biosensors because these devices can not only combine the microfluidic systems but also integrate into arrayed systems [60]. Moreover, the SPR platforms combined with surface modification approaches have been utilized for more advanced bioanalysis applications. For instance, Kurinomaru et al. successfully demonstrated that the SPR-based sensors rapidly analyzed the DNA methylation information by using synthetic immobilizer-based labeling for the selective condensation of the target DNA onto the sensor surface [61,62,63]. Judging from these facts, we believe that the SPR sensors decorated with the artificial materials have wide applicability for the detection of various molecules.

**Figure 10:**
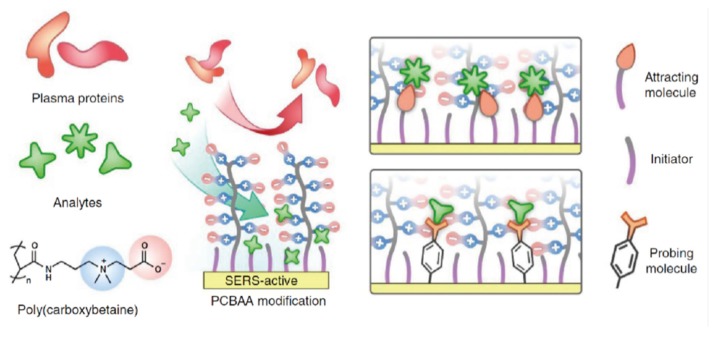
Schematic representation of the SERS-based protein sensor functionalized with the mixed SAM. Reproduced and adapted with permission from reference [44]. Copyright 2016 Springer Nature Limited.

Surface-enhanced Raman scattering spectroscopy (SERS) methods are also powerful approaches to determine the molecular recognition behavior of receptors at the interfaces with high sensitivity [64]. In SERS-based devices, the molecules captured on the rough surfaces of the metal substrates enhance the intensity of the inelastic scattering of photons (the Raman effect). Thus, the sensing behavior of synthetic receptor assemblies on the devices’ surfaces can be sensitively detected. Hung et al. reported real-time monitoring of drugs in blood plasma by using SERS-based devices functionalized with SAMs for chemical sensing [44]. In addition, the surface of the prepared SERS device was also coated by a zwitterionic poly(carboxybetaine) layer (PCBAA) to reduce the non-specific adsorption of plasma proteins (Figure 10). The drugs selected for this study were a tricyclic antidepressant (amitriptyline hydrochloride; AH) and anti-seizure medications (carbamazepine—CARB and phenytoin—PHEN), respectively. Mixed SAMs consisting of 3-mercaptopropionic acid and 1-propanethiol were utilized for the detection of the drug targets through electrostatic and hydrophobic interactions. As a result, the discrimination of these drugs in blood plasma was successfully demonstrated. More importantly, the prepared SERS sensors functionalized with the mixed SAMs showed high sensitivity in the presence of competing interferences (Table 1). The sensitivity achieved for the selected targets covered the clinically relevant levels of the drugs [65]. These results could be attributed to the effective interfacial design based on the mixed SAMs and the zwitterionic polymer layer [66]. Accordingly, the SERS sensors functionalized by the receptor assemblies are one of the powerful analytical tools for diagnosis applications.

**Figure 11:**
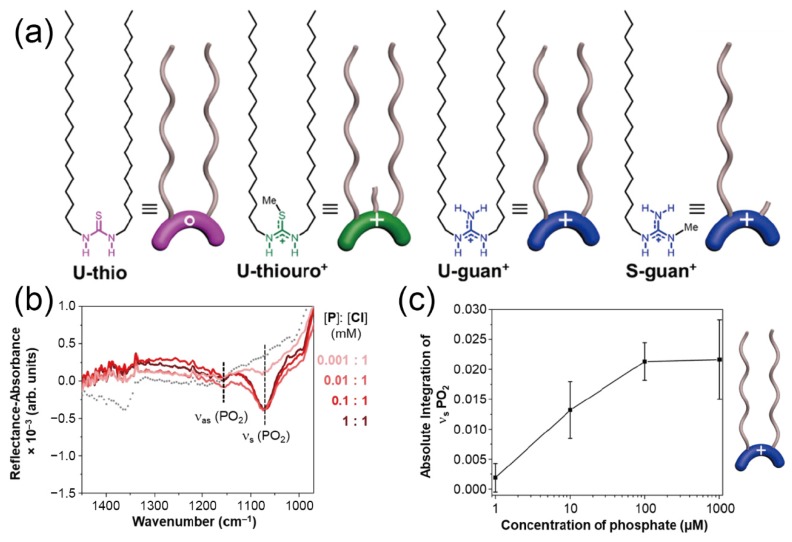
(**a**) Structures of a thiourea-based receptor (**U-thio**), a thiouronium-based receptor (**U-thiouro^+^**), and guanidinium-based receptors attached with the double-chain (**U-guan^+^**) and the single-chain (**S-guan^+^**). (**b**) IRRAS spectra of the **U-guan^+^** film upon the addition of analytes. (**c**) Changes in the absolute integration of the *ν*_s_(PO_2_) in the **U-guan^+^** film by adding phosphate. Reproduced and adapted with permission from reference [45]. Copyright 2019 American Chemical Society.

Moreover, infrared reflection–absorption spectroscopy (IRAS) methods were employed for minute analysis of the molecular recognition behavior of the sensing membranes at the interfaces [67]. In the IRAS platforms, the incident angle is set to around 80⁰ with respect to the vertical direction of the metal substrates. Thereafter, the infrared light is incident on the substrate and thus, the reflected light is detected. Hence, the IRAS methods can directly display the chemical information (i.e., molecular structures) of the captured analyte at the sensor surfaces, which means that the molecular interactions between the synthetic receptor membranes and the analytes can be quantitatively evaluated. Very recently, Flood and Allen et al. employed the IRAS method for investigating the sensing ability of an LB film for aqueous phosphates [45]. The LB film based on the guanidine moiety with the ‘double’ alkyl chain can optically respond to the addition of phosphate (Figure 11). Meanwhile, no optical response from the non-charged thiourea film was obtained upon the addition of phosphate. This suggests that the ability to recognize phosphate could be derived from the electrostatic interaction, which is enhanced at the interface (i.e., the air–water interface) [68]. Interestingly, the guanidinium moiety with the ‘single’ alkyl chain did not respond to the addition of phosphate. These results obtained for the IRAS study reveal that supramolecular organization (i.e., the higher-ordered structure of molecular assemblies) strongly affects the sensing ability of synthetic receptors at the interface.

### 4.3. Electrochemical/Electrical Detection of Analytes for the Achievement of On-site Quantitative Sensors

Although optical method-based chemical/biosensors exhibit high-sensitive responses to the addition of analytes, a large-sized and complicated equipment (e.g., spectrometer) is required for the quantitative determination of the analyte information. To achieve the on-site analyses of target species with high accuracy and quantitativity in the optical sensors, the handheld systems utilizing light-emitting diodes (LEDs)-based light sources combined with photodiodes or digital cameras are useful. However, the improvement of sensitivity in such compact measurement setup is generally difficult due to their low signal-to-noise ratio. Toward that end, electrochemical sensors are some of the more effective tools for the development of chemical analyzers utilizing synthetic receptors. For instance, cyclic voltammetry (CV) methods enable the sensitive detection of analytes. This is because a molecular complex, which consists of target species and sensing membranes on the sensing electrode, can be oxidized or reduced through electrochemical reactions at the specific potential [69]. For example, potentiometric methods using ion-selective electrodes (ISEs) and field-effect transistors (FETs) are directly able to transduce the molecular recognition phenomenon on the sensing electrodes to electrical detection signals (changes in the electrical potential) [70,71]. Therefore, external apparatuses are not required for the quantitative detection of analytes by using these types of sensors. Importantly, these kinds of electrical devices can easily integrate the sensor components sensing, signal-transducing, and data processing units) on one chip, suggesting that the electrochemical sensors contribute to the realization of high-throughput analysis platforms for various targets [72].

**Figure 12:**
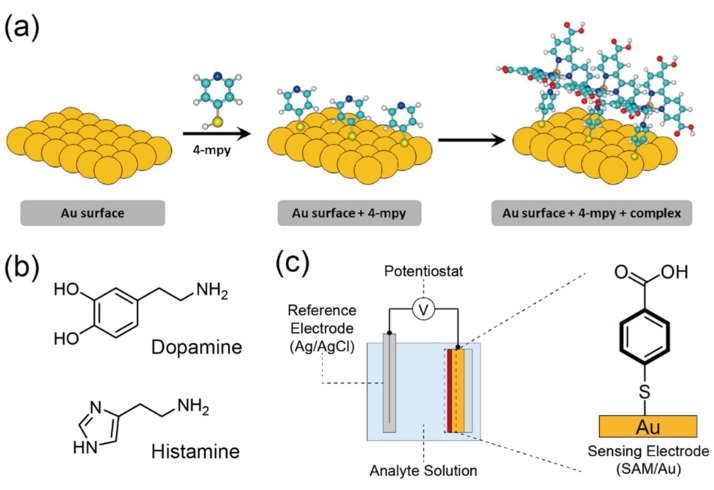
(**a**) The Ru(H_4_dcbpy)_2_(4mpy)-based SAM for the electrochemical detection of dopamine. Reproduced and adapted with permission from reference. [46]. Copyright 2019 Elsevier B. V. (**b**) Chemical structures of typical neurotransmitters. (**c**) Schematic illustration of the SAM-based potentiometric sensor for histamine. Reproduced with permission from reference [47]. Copyright 2019 The Royal Society of Chemistry.

The electrochemical detection of neurotransmitters (e.g., dopamine, histamine, etc.,) is an effective way to monitor nervous activity owing to their high-sensitivity [73]. However, it is hard to distinguish the required target from the other catecholamines electrochemically because the electroactive potentials of these molecules are very close. While accurate determination of the target neurotransmitter has been demonstrated by using enzyme-modified electrodes, [74] the utilization of enzymes makes it difficult to apply the prepared electrodes for the continuous measurement of nervous activity for a long time. This is derived from the chemical/physical instability of the naturally-derived molecular recognition materials (enzymes). In this regard, Bonacin et al. recently reported the selective detection of dopamine by a square wave voltammetry (SWV) measurement combined with a ruthenium-complex SAM (Figure 12a) [46]. In this study, a sensing electrode was initially modified by a 4-mercaptopyridine (4mpy) SAM. Then, ruthenium-complexed 2,2-bipyridine-4,4′-dicarboxylic acid [Ru(H_4_dcbpy)_2_] was made to react with the 4mpy-SAM on an Au electrode. The formed SAM [=Ru(H_4_dcbpy)_2_(4mpy)] had important features for the detection of dopamine on the electrode. First, Ru acts as the redox reactive site for enhancing the output current. Second, the dicarboxylic moiety can selectively recognize amine targets (i.e., dopamine) (Figure 12b) [75]. Based on these multiplier effects of the Ru(H_4_dcbpy)_2_-based SAM, relatively sensitive and selective detection of dopamine was performed by utilizing the electrochemical sensor. Furthermore, a SAM of 4-mercaptobenzoic acid (MBA) was also utilized for the achievement of sensitive and selective detection of another neurotransmitter (histamine) (Figure 12b) by using potentiometric measurements (Figure 12c) [47]. Although the sensitivity of potentiometric sensors is generally lower than that of other electrochemical methods (e.g., CV, SWV, etc.), the MBA-SAM-based sensor shows comparable sensitivity to that of other electrochemical sensors for histamine [76,77]. The sensitivity obtained might be derived from the complexation of histamine and the MBA molecule directly affecting the electric potential of the electrode through the conjugated backbone (benzene moiety) in the SAM [78]. Hence, electrochemical sensors functionalized with conjugated molecule-based SAMs can partially act like push–pull-type optical probes [79].

Field-effect transistors (FETs) can be employed as the transducers of potentiometric sensors because electrical characteristics in these semiconductor devices (output current and/or voltage) can directly reflect the changes in the electrical potential of the sensing electrodes [70]. More importantly, it is possible to construct the electronic circuits by using FETs as aforementioned. This means that external apparatuses are not required for the detection of analytes in FET-based sensors. Hence, FET devices are one of the best candidate platforms for the development of on-site chemical sensing systems [80]. However, amplification mechanisms for the analyte information should be introduced into the FET device because the sensitivity of the FET-based sensors is generally restricted by the Debye shielding effect [81]. In this regard, synthetic receptor membranes are considered as suitable materials to improve the sensing ability of the FET-based sensors due to their interesting features.

**Figure 13:**
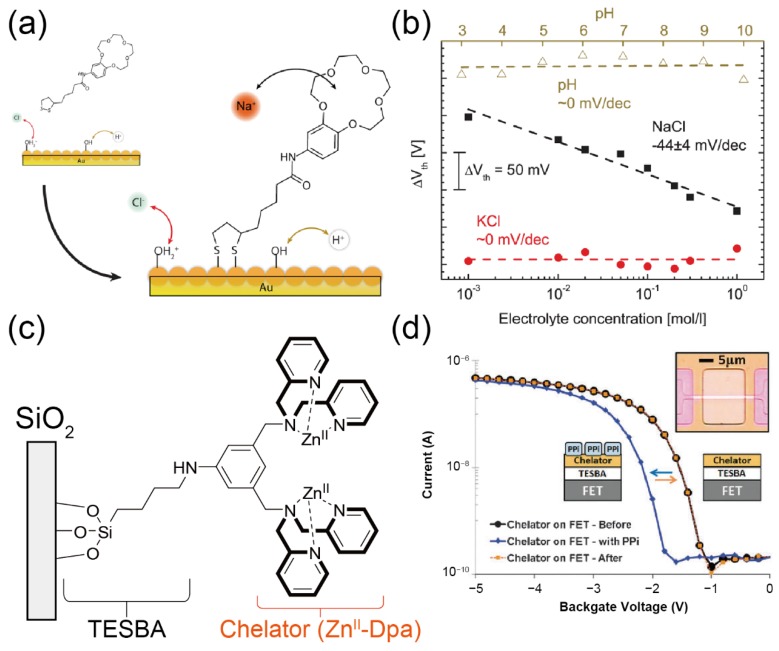
(**a**) Surface modification of the FET device with the 15-crown-5-ether monolayer for the detection of Na^+^. (**b**) Changes in threshold voltage (*V*_TH_) of the SAM-modified FET upon the addition of each electrolyte. Reproduced with permission from reference [48]. Copyright 2013 American Chemical Society. (**c**) Schematic representation of the Zn^II^-dpa moiety anchored with a 4-(triethoxysilyl)butyraldehyde (TESBA) monolayer on the FET device. (**d**) Transfer characteristics of the FETs before and after the functionalization of the gate surface by the Zn^II^-dpa SAM. Reproduced with permission from reference [49]. Copyright 2011 The Royal Society of Chemistry.

A gate electrode is usually utilized as a sensing portion in the FETs since the introduction of the molecular assemblies is easily achievable [80]. Wipf et al. reported the selective detection of sodium (Na^+^) by using a silicon nanowire FET modified with a 15-crown-5-ether monolayer [48]. The crown ether compound was functionalized with a dithiolane moiety to modify the gate electrode’s surface in the FET (Figure 13a). The crown ether-modified FET exhibited a highly selective response to the addition of Na^+^ in the presence of a competing interference (K^+^) (Figure 13b). The results obtained indicated that the selective detection of cations could be realized by utilizing the FETs modified with macrocyclic-based receptor membranes.

On the other hand, anion recognition in aqueous media is generally difficult due to strong hydration. In this regard, the FET-based anion sensors based on coordination bonding-driven receptors have been reported by several researchers. For example, Liu and Varma et al. demonstrated the electrical detection of phosphate anions utilizing the FET sensor decorated with a zinc-dipicolylamine (Zn^II^-dpa) SAM (Figure 13c) [49]. Because of the phosphate recognizing ability of the Zn^II^-dpa complexes [82], the electrical detection of pyrophosphate (PPi) was achieved on the Zn^II^-dpa-treated FET (Figure 13d). Based on the ability of the FET, prepared as above, to sense PPi, the electrical monitoring of DNA polymerase reactions was successfully demonstrated using the same device [50]. Interestingly, the slight changes in the molecular structures of phosphate anions were discriminated by the Zn^II^-dpa SAM [51]. This is because the assembled Zn^II^-dpa moiety in the SAM can elaborately respond to the condensed phosphoric acids (e.g., PPi, ATP, etc.,) through the multivalent molecular interactions [83].

**Figure 14:**
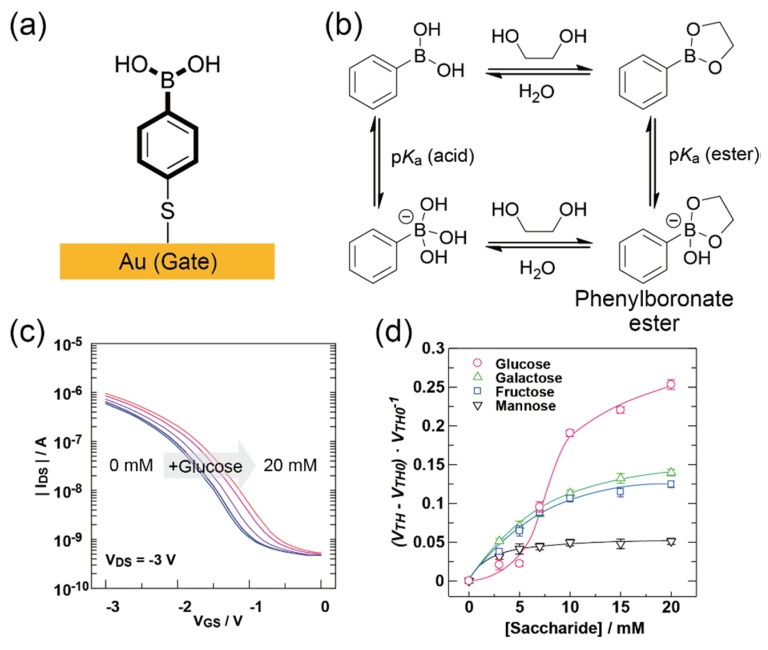
(**a**) Schematic structure of the PBA-SAM. (**b**) Multiple equilibria states involved in the cis-diol exchange with PBA. (**c**) Transfer characteristics of the PBA-modified FET upon the addition of glucose. (**d**) Changes in the output signal of the FET by the saccharides. Reproduced with permission from reference [52]. Copyright 2014 The Royal Society of Chemistry.

In general, electrochemical sensors including FETs detect electrical charges (electroactive sites) of analytes, suggesting that the electrical detection of non-charged molecules (e.g., saccharides) by FETs is still a challenging issue. To develop an FET-based sensor for non-charged saccharides, we employed a phenylboronic acid (PBA) SAM as the receptor membrane (Figure 14a) [52]. PBAs can recognize not only the cis-diol compounds including saccharides, but also the FET characteristics that are changed by the formation of negatively charged phenylboronate esters on the electrode (Figure 14b) [84]. As expected, the PBA-SAM attached FET responds to the addition of saccharides (Figure 14c). Importantly, the changes in the electrical signal of the device were exhibited as a sigmoidal response to the addition of glucose (Figure 14d). This might be attributed to the multi-site interactions between glucose and the PBA-SAM [85]. In fact, the electrical response to glucose was suppressed by decreasing the molecular density of PBA in the SAM, suggesting that the sensing ability of SAMs depends on the high-ordered structures of SAM molecules.

As a unique example of electrical signal-based chemical sensing, Sivalingam et al. demonstrated a light modulation system of output signals in metalloporphyrin-coated ZnO nanorod electrodes for the detection of cysteine [86]. In the proposed system, the interaction between the thiolated target (i.e., cysteine) and the metalloporphyrin derivative on the ZnO nanorod can be enhanced under visible light. The photo-induced modulation mechanisms of electrical properties in conductive materials could be explained by using the fundamental working principle of dye-sensitized solar cells [87]; the electron density in the porphyrin on the ZnO nanorod electrode might be decreased by irradiating the light source. Thus, the condensed level of cysteine onto the metalloporphyrin was increased, resulting that the enhancement of the reduction signal of cysteine on the electrode could be obtained. Significantly, such external-stimuli-induced signal amplification could be applied for various sensing platforms based on electrical devices functionalized with artificial materials. As stated previously, while the detection of macromolecular targets on electrochemical/electrical devices is not suitable (Table 2), detectable analytes in these sensors might be expanded by applying the signal amplification system.

### 4.4. Nanosensor Platforms Based on Other Physical Parameters

To study the molecular recognition information of synthetic receptor membranes, more physical parameters can also be utilized like the aforementioned optical or electrical signals. Quartz crystal microbalance (QCM)-type sensors are able to detect the “weight” of the captured analytes on the receptor layer [88]. The oscillation of quartz can be correctly controlled by applying an input signal through oscillator circuits because the quartz crystals exhibit inverse piezoelectric effects. Here, the oscillation frequency of the quartz crystal is changed by the number of molecules adsorbed (weight) on its surface. Hence, chemical sensing can be performed using QCM sensors. For instance, *p*-xylene in water was successfully detected by using the QCM sensor modified with a nickel-phthalocyanine (Ni^II^-pc) SAM [53]. The detection of aqueous phenols was also carried out on the QCM sensor functionalized with an LB film of Ni^II^-pc [54]. These sensors can distinguish tiny changes in the weight of the analytes from each other, which means that the QCMs decorated by synthetic receptors behave as molecular-leveled balances. While QCM sensors are not suitable for high-throughput analyses of the analytes as integration of QCMs into arrayed platforms is difficult, they are widely utilized as analytical tools in basic research; for example, the affinity between synthetic receptor materials and selected targets can be evaluated using QCM platforms [89].

Elastic waves at the interfaces decorated with receptor membranes have also been utilized as output signals for chemical sensing. The sensing mechanism of surface acoustic wave (SAW)-type sensors is as follows: the captured molecules on the surface of the SAW device functionalized with synthetic receptors affect the frequency of the SAW, suggesting that the shifts in the frequency reflect the sensing information of the receptors sensitively [90]. Although the detection of various gas molecules such as volatile organic compounds (VOCs) has been demonstrated using SAW-based sensors modified with molecular assemblies [55,91,92], the sensing of aqueous molecules utilizing the combination of SAW devices with synthetic receptors is still rare due to the low signal-to-noise ratio of SAW sensors in aqueous media [90]. Recently, Zhang et al. reported an amplification strategy for the output signal in the SAW-based sensor in the detection of biomolecules (exosomes) by using biomaterial-functionalized nanoparticles [93]. Thus, further developments of molecular assembly-functionalized SAW sensors can be expected.

## 5. Conclusions and Future Perspectives

In this review, we have provided an overview of the various chemical/biosensing platforms functionalized with synthetic receptor membranes ranging from the basic strategy for materials design to sensing applications. While synthetic receptors are effective materials to construct on-site sensing systems, these material-based sensors have never been popular for practical applications due to their low selectivity for the required targets as compared to biomaterials such as antibodies and enzymes. However, specific detection of the targets in organisms is achieved by integration of the molecular recognition moieties. Encouraged by this effective strategy for molecular recognition in nature, the bottom-up integration of synthetic receptors as the membranes at the interfaces has been employed for enhancing the sensing ability of artificial materials. The integration of molecular recognition moieties at the interfaces was successfully demonstrated by using the typical methods for the preparation of molecular assemblies, such as LB films or SAMs. The installation of the assemblies can be evaluated by various characterization techniques for the interfaces. The target selectivity of the synthetic receptor membranes was much higher than that of the single-molecule receptors because the driving forces for the target detection based on such materials are amplified at the interfacial fields. In addition, the presented molecular assemblies attached to the sensors are not only utilized as receptor functions, but these membranes can be also applied for the prevention of non-specific absorption of unintentional molecules onto the sensors [66]. Significantly, the concerted sensing features (e.g., multivalent molecular interactions) were confirmed in the molecular assembly-based sensors. Interestingly, these unique abilities obtained from the synthetic receptor membranes were manipulated by controlling the highly-ordered structures of the assemblies (i.e., the intermolecular distance of receptor moieties), suggesting that the sensing ability of the assembly-functionalized sensors can be easily fine-tuned without additional complicated synthesized materials. In fact, the relationship between the molecular recognition ability and lateral spacing of the molecular assembly at the sensing interfaces has been investigated by some groups [94,95,96]. From the perspective of materials science, it is interesting that the fact that the sensing features of molecules follow the configuration of molecular assemblies. Although we only presented the modification techniques for the preparation of monolayers or bilayers in this review, other coating techniques, such as layer-by-layer and chemical vapor deposition methods, can also utilized for the functionalization of sensing platforms [97]. Since the mentioned relationship between the sensing results and the nanostructures of the receptor membranes might be extended for various coating techniques, the sensing ability of sensors based on other modification techniques could be tuned by altering their coating conditions. In addition, the extraction effects of molecules in the fluidic systems might be utilized for the achievement of more amplification of the molecular recognition ability in the receptor membrane-attached surface of sensors [98]. Notably, the obtained results in the assembly-based sensors might reveal the more effective design of various artificial systems to us [99].

The sensitive detection of macromolecular targets (e.g., proteins) by using electrical devices is generally difficult, albeit their attractive features. Therefore, there is still room for the further development of biosensing devices by using other transduction mechanisms. However, we already proposed the bottom-up [100] or top-down approaches [101] for the amplification of the analyte information in the electrical device-based sensors. Hence, although various transducing mechanisms can be utilized for the building of molecular assembly-functionalized chemical/biosensors, we believe that electrical devices are one of the key platforms for the preparation of sensing systems for the on-site analyses of required target species owing to their quantitativity, easy fabrication, and compact integration.

From the viewpoint of supramolecular analytical chemistry, the fascinating point is that the modification of the sensing ability of the synthetic receptor assemblies can be easily accomplished. Recently, synthetic receptors combined with statistical analysis techniques, which are called chemosensor arrays, have been widely applied to achieve the simultaneous detection of multiple targets [102]. In a chemosensor array, the ‘meaningful’ cross-reactivity of the receptor library to various targets is important to realize the identification of analytes in the crude sample [103]. While precise design and complicated synthesis processes are generally required for controlling such cross-reactivity [104], the construction of sensors based on molecular assemblies might help to achieve simple processes for the preparation and design of sensor array systems without any complexity. More importantly, the synthetic receptor assemblies are very suitable for the functionalization of the device-based transducers as already mentioned. Accordingly, we believe that nanosensor platforms based on synthetic receptor assemblies will open up new avenues for the development of high-throughput molecular analyzers for the detection of various required targets that operate like next-generation genome sequencers.

## Figures and Tables

**Table 1:** Examples of molecular assembly-based nanosensors.

Synthetic Receptor	Type of Assembly	Transduction Mechanism	Analyte	LOD	Ref.
Thiourea	SAM	Colorimetry	Anions (CH_3_CO_2_^−^, H_2_PO_4_^−^, Cl^−^)	<0.1 mM	[40]
Thiourea (for anions), carboxylate (for cations)	SAM	Fluorometry	Inorganic anions (HSO_4_^−^, NO_3_^−^, H_2_PO_4_^−^, AcO^−^) or metal cations (Co^II^, Cu^II^, Ca^II^, Pb^II^)	<1 µM	[41]
Calix[4]crown	SAM	SPR	Potassium (K^+^)	1 pM	[42]
Zwitterions [carboxybetaine (CB), sulfobetaine (SB)]	SAM	SPR	Proteins (Albumin, IgG)	55.8 ng/mL	[43]
Carboxylate, propane	SAM	SERS	Drugs (CARB, PHEN, and AH)	0.5 µM (for CARB), 1 µM (for PHEN), 0.05 µM (for AH)	[44]
Guanidinium	LB film	IRAS	Phosphate	1 µM	[45]
Pyridine	SAM	CV	Dopamine	3.3 µM	[46]
Benzoic acid	SAM	Potentiometry (ISE)	Histamine	25 µM	[47]
15-Crown-5-ether	SAM	Potentiometry (FET)	Sodium (Na^+^)	NA	[48]
Zinc-dipicolylamine (Zn^II^-dpa)	SAM	Potentiometry (FET)	Pyrophosphate (PPi)	25 µM	[49,50]
Zinc-dipicolylamine (Zn^II^-dpa)	SAM	Potentiometry (FET)	Phosphates	NA	[51]
Phenylboronic acid (PBA)	SAM	Potentiometry (FET)	Saccharides	5 mM	[52]
Nickel-phthalocyanine (Ni^II^-pc)	SAM	QCM	*p*-Xylene	NA	[53]
Nickel-phthalocyanine (Ni^II^-pc)	LB film	QCM	Aqueous phenols	<1 mM	[54]
Trimethylchlorosilane	SAM	SAW	VOCs	<10 ppm	[55]

**Table 2:** “Typical” properties of presented transduction mechanisms in this review.

Transduction Mechanism	Example of Suitable Analytes	Miniaturization and Integration of Sensing System
Colorimetry	Ions, small molecules, peptides	Slightly difficult
Fluorometry	Ions, small molecules, peptides, proteins	Easy
SPR	Peptides, proteins	Very easy
SERS	Ions, small molecules	Difficult
IRAS	Ions, small molecules	Difficult
CV	Ions, small molecules	Easy
Potentiometry (ISE)	Ions, small molecules	Very easy
Potentiometry (FET)	Ions, small molecules, peptides	Very easy
QCM	Peptides, proteins	Difficult
SAW	Small molecules, peptides, proteins	Very easy

## Abbreviations

The following abbreviations are used in this review:

AFM	Atomic Force Microscope
ATR	Attenuated Total Reflection
BSA	Bovine Serum Albumin
CAG	Contact Angle Goniometry
CV	Cyclic Voltammetry
DPA	Dipicolylamine
ESCA	Electron Spectroscopy for Chemical Analysis
FET	Field-effect Transistor
FT-IR	Fourier Transform Infrared Spectroscopy
IRAS	Infrared Reflection-Absorption Spectroscopy
KFM	Kelvin Force Microscope
LB	Langmuir–Blodgett
LbL	Layer-by-Layer
LOD	Limit of Detection
MeCN	Acetonitrile
PBA	Phenylboronic Acid
PYS	Photoemission Yield Spectroscopy
QCM	Quartz Crystal Microbalance
SAM	Self-assembled monolayer
SAW	Surface Acoustic Wave
SERS	Surface-enhanced Raman Scattering
SPR	Surface Plasmon Resonance
STM	Scanning Tunneling Microscope
SWV	Square Wave Voltammetry
TAMRA	5-Carboxytetramethylrhodamine
TESBA	4-(Triethoxysilyl)butyraldehyde
UPS	UV Photoemission Spectroscopy
VOC	Volatile Organic Compound
XPS	X-ray Photoelectron Spectroscopy
